# Deep-Learning-Based Classification of Digitally Modulated Signals Using Capsule Networks and Cyclic Cumulants

**DOI:** 10.3390/s23125735

**Published:** 2023-06-20

**Authors:** John A. Snoap, Dimitrie C. Popescu, James A. Latshaw, Chad M. Spooner

**Affiliations:** 1 Department of Electrical and Computer Engineering, Old Dominion University, Norfolk, VA 23529, USA; jsnoa001@odu.edu (J.A.S.); jlats001@odu.edu (J.A.L.); 2 NorthWest Research Associates, Monterey, CA 93940, USA; cmspooner@nwra.com

**Keywords:** deep learning, digitally modulated signals, neural networks, signal classification, cyclostationarity

## Abstract

This paper presents a novel deep-learning (DL)-based approach for classifying digitally modulated signals, which involves the use of capsule networks (CAPs) together with the cyclic cumulant (CC) features of the signals. These were blindly estimated using cyclostationary signal processing (CSP) and were then input into the CAP for training and classification. The classification performance and the generalization abilities of the proposed approach were tested using two distinct datasets that contained the same types of digitally modulated signals, but had distinct generation parameters. The results showed that the classification of digitally modulated signals using CAPs and CCs proposed in the paper outperformed alternative approaches for classifying digitally modulated signals that included conventional classifiers that employed CSP-based techniques, as well as alternative DL-based classifiers that used convolutional neural networks (CNNs) or residual networks (RESNETs) with the in-phase/quadrature (I/Q) data used for training and classification.

## 1. Introduction

Blind classification of digitally modulated signals is a problem that occurs in both military and commercial applications such as signal intelligence, electronic warfare, or spectrum monitoring [1], and implementing conventional approaches to modulation classification in modern software-defined and cognitive-radio receivers can prove challenging [2]. These approaches use signal processing techniques and are grouped into two distinct classes:

- The first is likelihood-based methods [3,4], in which the likelihood function of the received signal is calculated under multiple hypotheses that correspond to the various signals that are expected to be received, and the classification decision is made based on the maximum of this function. We note that likelihood-based approaches are sensitive to variations in the signal parameters, which are expected to be estimated, and estimation errors can lead to significant performance degradation [5].
- The second is feature-based methods that use CSP techniques [6,7,8] in which CC features [9,10] are extracted from the received signal, and classification is accomplished by comparing the values of these features with prescribed values corresponding to the signals that are expected to be received [11]. As noted in [12,13], the performance of CC-based approaches to modulation classification is affected by the presence of multipath fading channels, and robust CC-based classifiers for multipath channels were discussed in [14,15,16].

As an alternative to the conventional methods mentioned above, in recent years, deep-learning (DL)-based techniques employing neural networks (NNs) have been explored for classifying signals using specific features that can be extracted from the data [17,18,19,20,21,22]. We note that NNs require extensive training to become proficient and that, depending on the type of NNs and features used, overfitting and a lack of generalization can affect the robustness of the trained NNs such that their performance degrades when presented with inputs that have different probability distributions than those in the training dataset. This aspect was studied in [23,24] in the context of DL-based approaches that use the I/Q components of the signal data to train different types of NNs such as CNNs, RESNETs, and CAPs for classifying signals, and it has been noted that NNs trained using the I/Q signal data have difficulty maintaining classification performance when presented with signals whose underlying digital modulation parameters have different probability distributions than those of the signals in the training dataset. This lack of generalization that affects the performance of NN classifiers that use the I/Q signal data is due to the fact that the I/Q components of digitally modulated signals are affected by various aspects with underlying random variables whose probability distributions widely vary in practice. These include the parameters of the digitally modulated signal such as the symbol interval or rate, the carrier frequency offset (CFO), the excess bandwidth, or the received signal power level, which will be discussed in more detail in subsequent sections, resulting in a virtually infinite set of possibilities for signal-generation parameters, so the dataset shift problem cannot simply be overcome by creating a large enough dataset of signals that accounts for all possible parameters of the digital modulation schemes to be classified. We note that the generalization aspect of DL-based classifiers for digitally modulated signals is an instance of domain adaptation, which is an important component of machine learning that deals with the ability of an NN trained in one or more *source domains* to transfer learning to a different (but related) *target domain* and has also been studied in the context of DL-based approaches for machine fault diagnosis [25,26], face recognition [27], or data analysis [28].

The above-mentioned aspects motivated the work presented in this paper, which proposes a new DL-based approach to the classification of digitally modulated signals with improved generalization abilities that employs CAPs in conjunction with the CC features of the signals. The synergistic use of CAPs and CC features is supported by the CAPs’ ability to distinguish digital modulations [29,30] and to outperform CNNs and RESNETs in the I/Q-based classification of digitally modulated signals [23], as well as by the proven robustness of the CC features obtained by CSP to co-channel signals [9,10] and to variations in noise models [31]. To the best of our knowledge, using CC features with DL-based signal classifiers has not been studied before. We note that this work was presented in part at the 2022 IEEE Military Communications Conference (MILCOM) [32] and that the current journal submission contains a more detailed study, which goes beyond that in [32] and includes: details on the CC features of digitally modulated signals and how these are estimated; a comparison of the proposed DL-based classifier with a conventional classifier that uses the CC features of digitally modulated signals as outlined in [10]. We also note the related approach in [33], which studied the use of a CNN in conjunction with the spectral correlation function (SCF) [6,7] in the context of spectrum sensing to classify wireless signals based on standards such as GSM, UMTS, and LTE and presented a comparison with alternative approaches used in spectrum sensing such as constant false alarm rate (CFAR) detectors or support vector machines (SVMs). However, the use of the SCF alone is not sufficient to distinguish between the various MPSK (M≥4) and QAM signals considered in the processed datasets herein, as those signals have identical SCFs.

In this context, our goal was to provide full details on the use of CAPs and CC features for signal classification and to assess the robustness and generalization abilities of the proposed DL-based digital modulation classifier using CAPs and CCs on two distinct datasets that are publicly available from [34]. These datasets include signals with similar digital modulation schemes, but which were generated using distinct parameters, and are suitable for generalization studies as data from one dataset can be used for training while data from the other dataset are used for testing. Furthermore, we also compared the proposed classifier with a baseline classification model in which the cycle frequencies corresponding to non-zero CCs were extracted using CSP and used for signal classification, as well as with the alternative DL-based classifier in [23], which uses a CAP with the I/Q signal data for classification.

The remainder of this paper is organized as follows: Section 2 provides a brief review of the CC features for digitally modulated signals and introduces the baseline classification model. This is continued in Section 3 with the presentation of the proposed DL-based approach to digital modulation classification, which involves the use of CAPs in conjunction with the CC features of digitally modulated signals for training and signal classification. Section 4 describes the datasets used for CAP training and performance evaluation and is followed by the presentation of the numerical results and the performance analysis in Section 5. A brief discussion of the results and suggestions for further investigation are provided in Section 6, and the paper is concluded with final remarks in Section 7.

## 2. CC Features for Digitally Modulated Signals and Baseline Classification Model

CSP provides a set of analytical tools for estimating distinct features that are present in various modulation schemes and that can be used for blind signal classification in various receive scenarios, which include stationary noise and/or co-channel interference. These tools enable the estimation of higher-order CCs [9,10] and of the SCF [6,7] from the received signal, such that the estimates of the CC or of the SCF can then be compared to a set of theoretical values of the CCs or the SCF, for classifying the corresponding digital modulation scheme embedded in the received signal. We note that, in addition to being affected by propagation, which includes noise and/or co-channel interference, the received signal features are also affected by other aspects with underlying random variables whose probability distributions vary widely in practice and influence the decisions of blind classifiers. These may include (but are not limited to): the symbol interval or the corresponding data rate, the carrier frequency offset (CFO), the excess bandwidth of the signal implied by variations of the pulse-shaping function parameters, or the received signal power level, which directly impacts the in-band signal-to-noise ratio (SNR). While some of these parameters that affect signal characteristics may have limited practical ranges, such as the roll-off parameter β in the case of square-root raised-cosine (SRRC) pulse-shaping for example, which is typically in the [0.2,0.5] range, others such as the symbol interval or the CFO possess an infinite number of valid practical choices.

We emphasize here that CCs, although conceptually and mathematically obscure and complex, are intimately related to the set of *n*th-order probability density functions (PDFs) governing the behavior of communication signals [6,7]. As outlined in Section 2.1, CCs are the Fourier series components of the power-series-expansion coefficients of the logarithm of the characteristic function, which itself is simply the Fourier transform of a PDF. CCs are not the sort of features that are typically associated with machine learning and data mining, where voluminous datasets are searched with fast computers for correlations between mathematical transformations of the data and the signal-class label. Being strongly related to all joint PDFs associated with the signals’ samples, the use of CCs as features has much more in common with decision-theoretic approaches than with modern feature-based approaches, for which there may not be any provided mathematical rationale [11,35].

### 2.1. CC Feature Extraction

Consider the generic digitally modulated signal written as:

(1) $$\begin{array}{c} x\left(t\right)=as\left(t\right)e^{i(2\pi f_{0}t+\phi )}+w\left(t\right), \end{array}$$

where *a* denotes the signal amplitude, $s\left(\cdot \right)$ is the complex envelope of the signal, $f_{0}$ is the CFO, and $w\left(t\right)$ is additive white Gaussian noise (AWGN). The CC features of this signal are extracted using a CSP-based approach [9,10,11] that starts with the *n*th-order temporal moment function defined by

(2) $$\begin{array}{c} R_{x}\left(t,\tau ;n,m\right)={\hat{E}}^{\left\{\beta \right\}}\left\{\prod_{j=1}^{n}x^{\left(*\right)j}\left(t+\tau_{j}\right)\right\}, \end{array}$$

where *m* of the factors are conjugated, $\left(*\right)$ represents an optional conjugation, and ${\hat{E}}^{\left\{\beta \right\}}$ is the multiple sine-wave extraction operator, which is the direct analog of the stochastic process expected value operator in the fraction-of-time probability framework [6],

(3) $$\begin{array}{c} {\hat{E}}^{\left\{\beta \right\}}\left\{g\left(t\right)\right\}=\sum_{\beta }g_{\beta }e^{i2\pi \beta t}, \end{array}$$

for arbitrary g(t), with

(4) $$\begin{array}{c} g_{\beta }≜\lim_{T\to \infty }\frac{1}{T}\int_{\frac{-T}{2}}^{\frac{T}{2}}g\left(u\right)e^{-i2\pi \beta u}\;du\equiv \langle g\left(u\right)e^{-i2\pi \beta u}\rangle , \end{array}$$

and the summation in (3) is taken over all β for which $g_{\beta }\neq 0$. The corresponding *n*th-order temporal cumulant function (TCF) is given by:

(5) $$\begin{array}{c} C_{x}\left(t,\tau ;n,m\right)=\sum_{P_{n}}\left[h\left(p\right)\prod_{j=1}^{p}R_{x_{\nu_{j}}}\left(t,\tau_{\nu_{j}};n_{j},m_{j}\right)\right], \end{array}$$

where the sum is over all distinct partitions ${\left\{\nu_{j}\right\}}_{j=1}^{p}$ of the index set $\left\{1,2,\ldots ,n\right\}$ and $h\left(p\right)={\left(-1\right)}^{p-1}\left(p-1\right)!$. We note that the *n*th-order moment functions are polyperiodic functions of time, which implies that the *n*th-order cumulant functions are as well, and thus, each of them can be represented in terms of a generalized Fourier series.

The coefficients of the cumulant Fourier series represent the CCs of the signal (1) and are given by [9]

(6) $$\begin{array}{c} C_{x}^{\alpha }\left(\tau ;n,m\right)=\langle C_{x}\left(t,\tau ;n,m\right)e^{-i2\pi \alpha t}\rangle , \end{array}$$

where α is an *n*th-order cycle frequency (CF) of the signal.

The CFs α for which the CC (6) is not zero for typical digitally modulated signals include the harmonics of the symbol rate $1/T_{0}$, multiples of the CFO $f_{0}$, and combinations of these two sets, such that α can be written as

(7) $$\alpha =\left(n-2m\right)f_{0}\pm k/T_{0}.$$

For second-order CFs (n=2), the non-conjugate CFs corresponding to m=1 depend only on the symbol rate $1/T_{0}$, while the conjugate CFs corresponding to m=0 depend on both the symbol rate $1/T_{0}$ and the CFO $f_{0}$.

To obtain accurate estimates of the CC features for signal classification, knowledge of signal parameters such as the symbol rate and CFO is necessary, as these parameters define the CFs needed for CC computation. Estimates of these signal parameters are obtained using CSP techniques such as the strip spectral correlation analyzer (SSCA) [36] or the time- and frequency-smoothing methods in [6,7] and can be further refined using additional parameters such as the excess bandwidth and the in-band SNR, which can be estimated using energy-based band-of-interest (BOI) detectors that do not require CSP [37].

To extract the necessary signal parameters for CC estimates, we used this procedure:

1. Use the BOI detector [37] to evaluate the signal bandwidth and obtain a low-resolution estimate of the center frequency.
2. Frequency shift the BOI to the baseband using the low-resolution CFO estimate.
3. Downsample/upsample the data as necessary such that the signal bandwidth is maximized, but keep the fractional bandwidth of the result strictly less than 1.
4. Apply the SSCA to the data provided by Step 3 to detect the second-order CFs.
5. Use the non-conjugate second-order CFs (if these are present) to obtain a high-resolution estimate of the symbol rate $1/T_{0}$.
6. If no non-conjugate CFs are present, the symbol rate may be estimated from any conjugate CFs present, which can also be used to provide a high-resolution estimate of the CFO.
7. Determine the basic pattern of the second-order CFs present in the BOI.
8. If conjugate CFs are not present from Step 4, then the data from Step 3 are raised to the fourth power and Fourier transformed, and further CSP is applied to determine the CF pattern and to estimate the symbol rate if not provided by Step 5 and to obtain a high-resolution estimate of the CFO.

**Figure 1:**
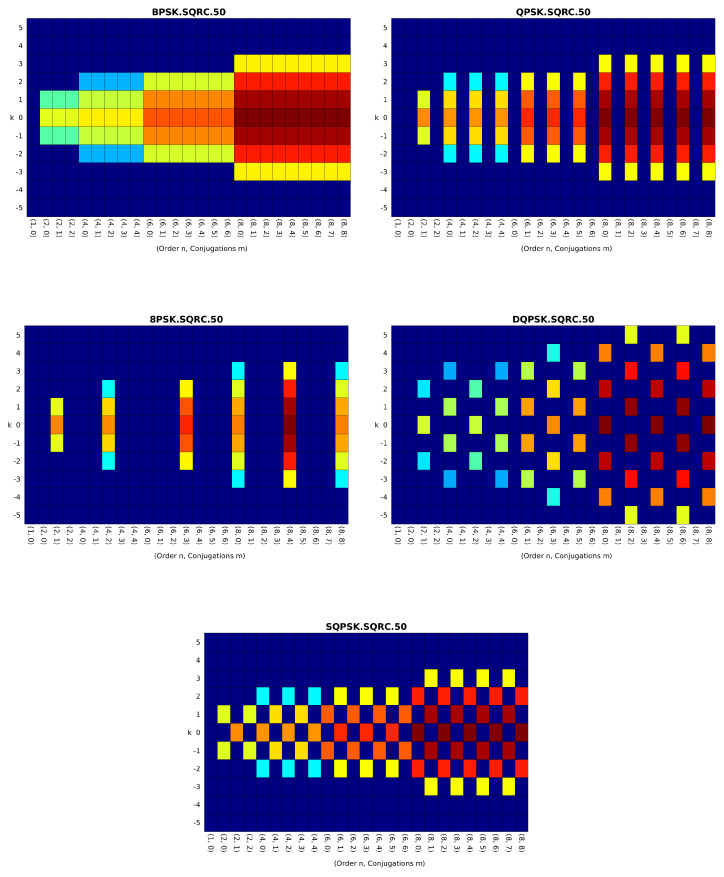
Five common CF patterns for digital QAM and PSK modulation types. The CF pattern is more general than the constellation, with the shaping pulse bandwidth controlling the number of significant CC values in the harmonic number (*k*) dimension, and the probabilistic structure of the symbol random variable controls the pattern across the (n,m) dimension [7].

We note that Steps 7–8 are a key element of the procedure, which aims at identifying the basic second-order CF pattern of the signal implied by (7), which for typical digital modulation schemes is one of the following: BPSK-like, QPSK-like, π/4-DQPSK-like, 8PSK-like, and staggered QPSK (SQPSK)-like. Furthermore, all digital QAM signals with balanced (symmetric) constellations and more than two constellation points map to the QPSK-like pattern and amplitude-shift-keyed signals, BPSK, and OOK map to the BPSK-like patterns. For an illustration, some of these CF patterns are shown in Figure 1.

Once the CF pattern is identified, one can also determine the actual number of CFs needed to fully characterize the modulation type through its set of associated CC values.

To reduce computations for the CCs (6), we used the following parameters:

- The delay vector τ=0;
- The orders of CC features were limited to the set $n=\left\{2,4,6\right\}$, and the number of conjugation choices was constrained by the order *n* to n+1;
- For each $\left(n,m\right)$ pair, the CFs where CCs are non-zero are related to the CFO ($f_{0}$) and symbol rate ($1/T_{0}$) by Equation (7), where the set of non-negative integers *k* is restrained to a maximum value of five.

These settings imply a total of 11 potential CFs for each of the 15 $\left(n,m\right)$ pairs or a maximum of 165 CC estimates for each digitally modulated signal to be classified. The actual number will depend on the blindly estimated second-order CF pattern.

### 2.2. The Cyclic Cumulant Estimate

After the band-of-interest detection, blind key parameter estimation, and CF pattern determination are performed, the cyclic cumulants can be estimated. This was performed by combining appropriate estimates of cyclic temporal moment functions (CTMFs), which are the Fourier series coefficients of the temporal moment functions (2). The CTMF for cycle frequency β is given by

(8) $$\begin{array}{c} R_{x}^{\beta }\left(\tau ;n,m\right)=\lim_{T\to \infty }\frac{1}{T}\int_{-T/2}^{T/2}R_{x}\left(t,\tau ;n,m\right)e^{-i2\pi \beta t}\;dt. \end{array}$$

It can be shown that the cyclic cumulant (6) is given by

(9) $$\begin{array}{c} C_{x}^{\alpha }\left(\tau ;n,m\right)=\sum_{P_{n}}\left[h\left(p\right)\sum_{\beta 1^{†}=\alpha }\prod_{j=1}^{p}R_{x_{\nu_{j}}}^{\beta_{j}}\left(\tau_{\nu_{j}};n_{j},m_{j}\right)\right], \end{array}$$

where $\beta =[\beta_{1}\;\beta_{2}\ldots \beta_{p}]$, and the sum over β requires the inclusion of all such distinct CTMF cycle frequency vectors whose components sum to the target cyclic cumulant cycle frequency α [6,7].

An estimate of the CC (9) (equivalently (6)) is given by simply replacing the infinite-time averages in the definition of the Fourier coefficients (8) with finite-time averages and by replacing the TMF in (8) with the corresponding homogenous delay product:

(10) $$\begin{array}{c} {\hat{R}}_{x}^{\beta }\left(\tau ;n,m\right)=\frac{1}{T}\int_{0}^{T}\left[\prod_{j=1}^{n}x^{{\left(*\right)}_{j}}\left(t+\tau_{j}\right)\right]e^{-i2\pi \beta t}\;dt, \end{array}$$

where the shift in the integration interval results in a phase shift of the CTMF relative to (8) and is easily accommodated. The estimate of the cyclic cumulant is then given by the properly weighted sum of products of CTMF estimates:

(11) $$\begin{array}{c} {\hat{C}}_{x}^{\alpha }\left(\tau ;n,m\right)=\sum_{P_{n}}\left[h\left(p\right)\sum_{\beta 1^{†}=\alpha }\prod_{j=1}^{p}{\hat{R}}_{x_{\nu_{j}}}^{\beta_{j}}\left(\tau_{\nu_{j}};n_{j},m_{j}\right)\right]. \end{array}$$

Because we have estimated the CF pattern and the key signal parameters, we can find all required lower-order cycle frequencies implied by the sum over the lower-order cycle frequency vector β.

### 2.3. Baseline Classification Model

Our baseline classification model was a conventional CC-based classifier as outlined in [10], where the CC values were estimated for each signal to be classified and a modulation classification decision was made based on the closest proximity of the estimated CCs to a modulation’s theoretical CC values to assign a signal modulation label as discussed in [10]. Eight digital modulation schemes of interest were considered: BPSK, QPSK, 8PSK, π/4-DQPSK, MSK, 16QAM, 64QAM, and 256QAM, and the performance of the baseline classification model was assessed on the two datasets available from [34]. Details on these datasets are discussed in Section 4, and we emphasize that all of the signal processing techniques used in our baseline classification model were fully blind and all parameters needed (such as the symbol interval or rate, CFO, or signal bandwidth) were estimated from the I/Q data using signal processing as outlined in Section 2.1. This allowed a fair comparison with the performance of the proposed DL-based classifier, which uses capsule networks, which is described in the following section, because this was also not provided any prior information when making an inference.

## 3. Cyclic Cumulants and Capsule Networks for Digital Modulation Classification

CAPs are a particular set of NNs that have been introduced in the context of emulating human vision [38] because of their proven ability to focus on learning desirable characteristics of the input pattern or signal, which correspond to a specific input class. In the case of the human visual system, when the eye is excited by a visual stimulus, it does not focus on all available inputs, but rather establishes points of fixation instead, which can be thought of as characteristics present in the input data that are useful for classification. CAPs are a special class of shallow CNNs in which the learned desirable characteristics of the training dataset are captured by means of capsules consisting of multiple parallel and independent units that can learn class-specific characteristics of the training data. CAPs differ from CNNs, which rely on a single output neuron per class, as well as from multi-branch NNs, in which the multiple branches processed are recombined into fully connected layers.

**Figure 2:**
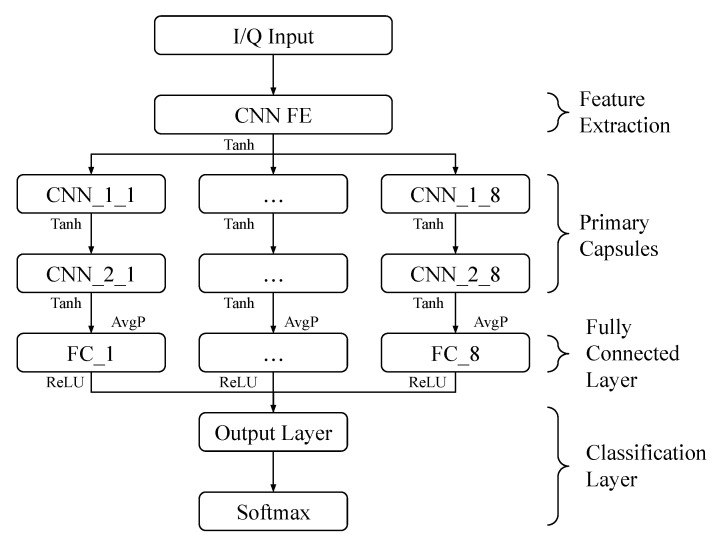
CAP with eight branches for classifying the eight digital modulation schemes of interest.

In recent years, CAPs have been successfully used in DL-based modulation recognition systems [29,30] and have been shown to display better classification performance than CNNs and RESNETs in the I/Q-based classification of digitally modulated signals [23]. The apparent superiority of CAPs over other types of NNs has prompted our proposed approach for digital modulation classification, in which we used the CC features of digitally modulated signals as the inputs to the CAP to train it to classify the same eight digital modulation schemes of interest mentioned in Section 2.3 (BPSK, QPSK, 8PSK, π/4-DQPSK, MSK, 16QAM, 64QAM, and 256QAM). Consequently, the CAP used in our approach consists of eight capsules, as illustrated in Figure 2, taking as inputs the 11×15=165 CC values of the received signal, which matches the dimension of the input layers for the defined capsules.

Because in general, the higher-order CCs have larger magnitudes than the lower-order CCs and because the CCs also scale with the signal power, the CC estimates ${\hat{C}}_{x}^{\alpha }\left(\tau ;n,m\right)$ were further processed as follows prior to use with the proposed CAP for training and classification:

- *Warping*: This involves using the order *n* of the CC estimates to obtain “warped” versions ${\hat{C}}_{x}^{\alpha }{\left(\tau ;n,m\right)}^{\left(2/n\right)}$. We note that CSP-based blind modulation classification also employs warped CC estimates.
- *Scaling*: The warped CC estimates were subsequently scaled to a signal power of unity, using a blind estimate of the signal power. This provided consistent values for the capsule network to train on and prevented varying signal powers from causing erroneous classification results due to neuron saturation—a common issue with input data that do not go through some normalization process.

After these pre-processing steps, the warped and scaled CC estimates can be used to train the CAP with the structure shown in Figure 2 and parameters outlined in Table 1, and subsequently, the trained CAP can be employed to blindly classify digitally modulated signals. The various components of the proposed CAP include:

- Feature extraction layer: This first layer of the network performs a general feature mapping of the input signal, and its parameters are similar to those used in other DL-based approaches to classification of digitally modulated signals [21,24,39]. This layer includes a convolutional layer followed by a batch normalization layer and an activation function.
- Primary capsules: This layer consists of eight primary capsules, which is equal to the number of digital modulation classes of interest. These capsules operate in parallel using as the input the output from the feature extraction layer, and each primary capsule includes two convolutional layers with a customized filter, stride, and activation function, followed by a fully connected layer.
- Fully connected layer: This layer consists of a 1×8 neuron vector with the weights connecting to the previous layer. Each neuron in the last layer of the primary capsules will be fully connected to each neuron in this layer. These neurons are expected to discover characteristics specific to the capsules’ class. To make the output of the network compatible with a SoftMax classification layer, each neuron within this layer is fully connected to a single output neuron, and the output neurons for all primary capsules are combined depthwise to produce an eight-dimensional vector **n**, which is passed to the classification layer. The value of each respective element of **n** will be representative of the likelihood that its corresponding modulation type is present in the received digitally modulated signal.
- Classification layer: In this layer, vector **n** is passed to the SoftMax layer, which will map each element $n_{i}$, i=1,…,8, in **n** to a value:
  (12) $$\sigma_{i}\left(n\right)=\frac{e^{n_{i}}}{\sum_{j=1}^{8}e^{n_{j}}},$$
  where $\sigma_{i}\left(n\right)\in \left[0,1\right]$, with each element representing the probability of occurrence, such that the sum of elements in **n** adds up to 1 [40]. This provides a convenient way to determine which modulation type is most likely to correspond to the signal at the input of the CAP.

We note that, similar to the CAP used in [23], the CAP described above was inspired by [38], and its structure and topology were established using a custom genetic algorithm that determined the CAP parameters shown in Table 1 (convolutional layer filter size, filter stride, and the number of layers). While providing full details of the genetic algorithm employed is beyond the scope of the paper, we mention that the algorithm would randomly choose a value (over a defined interval) for each of the above-listed parameters, randomly turning off a layer or adding a new one. This “pseudo-randomly” generated network would then be trained and evaluated against the testing dataset, repeating the experiment multiple times, with the best-performing networks being noted. The hyper-parameters of the best-performing network(s) were noted, and their likelihood of selection for subsequent experiments was slightly increased. Over the course of many such experiments, networks having a specific layer structure and layer hyper-parameters began to emerge, which helped to inform the chosen topology.

## 4. CAP Training and Performance Evaluation

To train and assess the modulation classification performance of the proposed CAP (including its out-of-distribution generalization ability), we used two datasets that both contain the eight modulation types of interest (BPSK, QPSK, 8PSK, π/4-DQPSK, MSK, 16QAM, 64QAM, and 256QAM) and are publicly available from [34].

### 4.1. The Training/Testing Datasets

The two datasets are referred to as CSPB.ML.2018 and CSPB.ML.2022 [34], and details about their signal-generation parameters are given in Table 2. To summarize their characteristics, we note that each of the datasets contains collections of the I/Q data corresponding to a total of 112,000 synthetic digitally modulated signals that include equal numbers of the eight digital modulation schemes of interest. With the exception of MSK-modulated signals, all other signals employ square-root raised-cosine (SRRC) pulse-shaping with roll-off factor β, and 32,768 samples for each instance of each signal are provided. We note that the listed SNRs for the signals in both datasets correspond to in-band SNR values and that a BOI detector [37] was used to validate the labeled SNRs, CFOs, and SRRC roll-off values for the signals in both datasets.

Reviewing the signal-generation parameters of these two datasets outlined in Table 2 confirmed that they are suited for testing the generalization abilities of the proposed capsule network as the signals in the two datasets were generated with distinct non-overlapping ranges for the CFO. Specifically, the maximum CFO in CSPB.ML.2018 is 0.001, while the minimum CFO in CSPB.ML.2022 is an order of magnitude larger at 0.01. We note that, as the other signal-generation parameters are similar for the two datasets, the differences in the CFO will enable the observation of the generalization abilities of the trained CAPs, as will be discussed in detail in Section 5:

- For the CAP that uses the I/Q signal data for training and testing, the CFO shift in the testing dataset relative to the training dataset resulted in significant degradation of the classification performance of the CAP and indicated that it was unable to generalize its training to new datasets that contain similar types of signals, but with differences in some of their digital modulation characteristics. This aspect was also reported in [23], and similar results have been reported for CNNs and RESNETs in [24].
- As will be seen in Section 5, the CAP that uses the CC features for training and testing the CFO shift in the testing dataset relative to the training dataset resulted in similar classification performance and indicated that the CAP trained using CC features was resilient to variations of the CFO from the training dataset.

### 4.2. CAP Training

The proposed CAP was implemented in MATLAB and trained on a high-performance computing cluster with 18 NVidia V100 graphical processing unit (GPU) nodes available, with each node having 128 GB of memory. We note that, while the DL network training process is computationally intensive, if the available computing resources are leveraged appropriately such that the entire training dataset is loaded into the available memory, training can be completed in several minutes for the CC-trained networks (provided the CC estimates are readily available), as compared to several hours in the case of a CAP that uses the I/Q signal data [23]. The CC-trained CAPs obtained the best results with an adaptive moment estimation (Adam) optimizer [41] using ten epochs while shuffling the training data before each training epoch, a mini-batch size of 250, an initial learn rate of 0.001, a piecewise learning schedule involving a multiplicative learning rate drop factor of 0.9 every five epochs, an $L_{2}$ regularization factor of 0.0001, a gradient decay factor of 0.9, a squared gradient decay factor of 0.999, an epsilon denominator offset of $10^{-8}$, and a final batch normalization using the entire training data population statistics.

**Figure 3:**
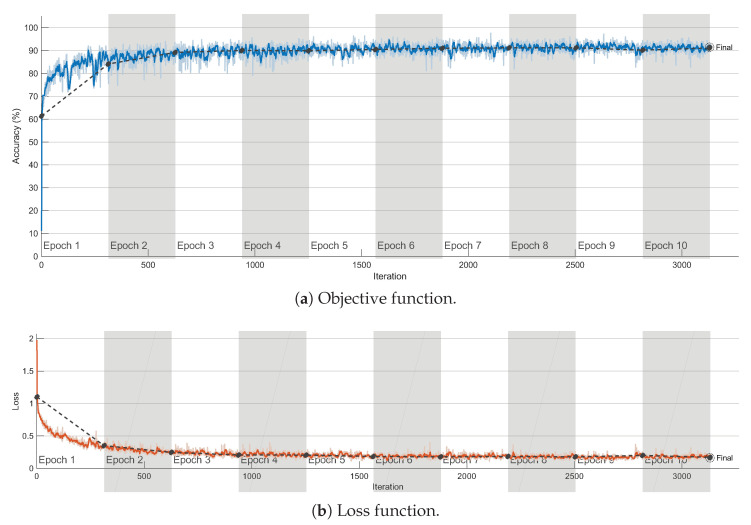
Objective and loss functions for the CC-trained CAP using the CSPB.ML.2018 dataset.

Two distinct training/testing instances were performed as follows:

- In the first training instance, dataset CSPB.ML.2018 was used, splitting the available signals into 70% for training, 5% for validation, and 25% for testing. The corresponding objective and loss functions for the trained CAP are shown in Figure 3, and we note that the probability of the correct classification for the test results was obtained using the 25% test portion of the signals in CSPB.ML.2018.
       The CAP trained on CSPB.ML.2018 was then tested on dataset CSPB.ML.2022 to assess the generalization abilities of the trained CAP in classifying all signals available in CSPB.ML.2022.
- In the second training instance, the CAP was reset and trained anew using the signals in dataset CSPB.ML.2022, with a similar split of 70% of signals used for training, 5% for validation, and 25% for testing. The corresponding objective and loss functions for the trained CAP were similar to the ones in Figure 3 and were omitted for brevity. The probability of correct classification for the test results was obtained using the 25% test portion of the signals in CSPB.ML.2022.
       The CAP trained on CSPB.ML.2022 was then tested on dataset CSPB.ML.2018 to assess the generalization abilities of the re-trained CAP when classifying all signals available in CSPB.ML.2018.

### 4.3. Assessing Generalization Abilities

We note that the CFOs for both datasets were generated randomly with a uniform distribution and that the CFO distribution interval for CSPB.ML.2018 was non-intersecting with the CFO interval for CSPB.ML.2022. This was performed to assess the ability of a trained CAP to generalize:

- If the CAP was trained on a large portion of CSPB.ML.2018 and its performance when classifying a remaining subset of CSPB.ML.2018 was high, but its performance when classifying CSPB.ML.2022 was low, then the CAP’s ability to generalize was low, and its performance was vulnerable to shifts in the signal parameter distributions.
- By contrast, if the classification performance of the CAP on both the remaining subset of CSPB.ML.2018 and on all of CSPB.ML.2022 was high, then its generalization ability was high, and the CAP was resilient to shifts in signal parameter distributions.

## 5. Numerical Results and Performance Analysis

**Figure 4:**
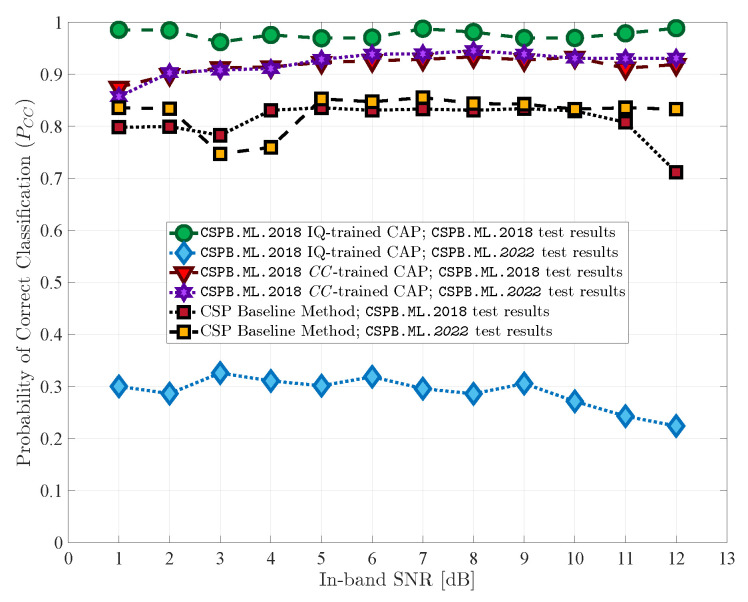
Initial and generalization test results for CAPs trained on CSPB.ML.2018. The CC-trained CAP exhibited high classification performance under both testing scenarios.

**Figure 5:**
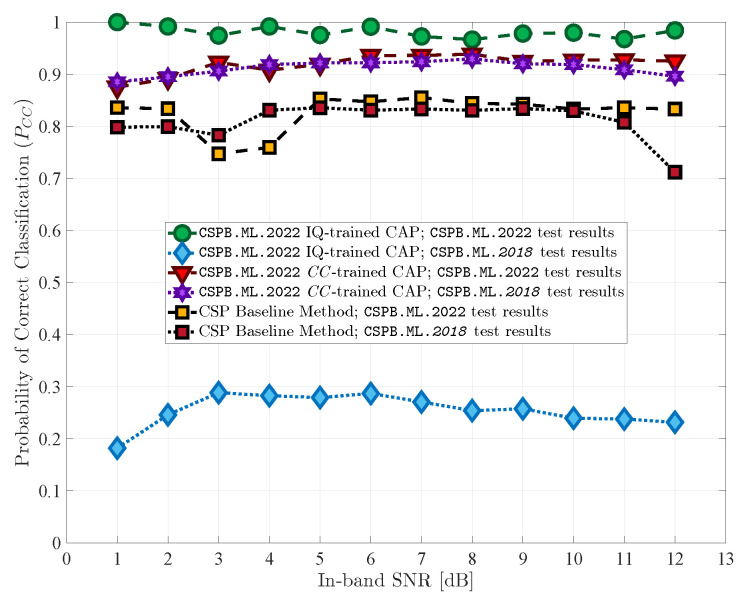
Initial and generalization test results for CAPs trained on CSPB.ML.2022. The CC-trained CAP exhibited high classification performance under both testing scenarios.

We ran simulations to test the classification performance of the proposed CAP and compared it to the performance of the baseline classification model and to the performance of a CAP that used I/Q data as the input [23]. The results are summarized in Table 3 and Figure 4 and Figure 5 and are discussed in the following sections.

### 5.1. Baseline Model Performance

The baseline classification model requires no training as it was based solely on comparing the estimated CCs of the digitally modulated signal with theoretical CC values corresponding to different modulation schemes. This conventional CSP approach to the classification of digitally modulated signals resulted in good performance for both datasets, CSPB.ML.2018 and CSPB.ML.2022. By utilizing all of the 32,768 samples for each signal in a dataset, the baseline model yielded quite accurate estimates of the CC features and resulted in an overall modulation classification accuracy of 82.0% for both CSPB.ML.2018 and CSPB.ML.2022.

We note that the baseline performance curves shown in Figure 4 and Figure 5 do not display a steady increase in $P_{CC}$ with increasing SNR, which highlights the difficulty associated with performing modulation classification on these two datasets CSPB.ML.2018 and CSPB.ML.2022 using conventional CSP-based approaches. Nevertheless, the results of the baseline model set a clear standard for performance and generalization because it was based on CSP and the minimum distance to theoretical CCs, with no training required, such that the classification results can be obtained with equally high performance, independent of signal parameter distribution functions.

### 5.2. CC-Trained CAP Performance

For the proposed CAP trained using CC features extracted from signals in dataset CSPB.ML.2018, the variation of $P_{CC}$ versus the SNR is shown in Figure 4. The overall $P_{CC}$ achieved by this network was 92.3%, which was about a 10% improvement over the baseline model. When the CAP trained using CC features from signals in the CSPB.ML.2018 dataset was used to classify signals in the CSPB.ML.2022 dataset, the overall $P_{CC}$ value continued to remain high at 93.1%, which was about 11% larger than that of the baseline classification model and implied excellent generalization abilities.

When the CAP was trained using CC features from the signals in the CSPB.ML.2022 dataset, the variation of $P_{CC}$ versus SNR is shown in Figure 5, and its classification performance was similar to the previous case. The overall $P_{CC}$ achieved by this network was 92.5%, which was again about a 10% improvement over the baseline model. Furthermore, the CAP was able to generalize and maintained an overall $P_{CC}$ value of 91.6% when tested with signals in the CSPB.ML.2018 dataset.

These results showed that the proposed CAP can be successfully trained using CC features to perform modulation classification with an overall $P_{CC}$ that exceeded that of conventional CSP-based classification approaches, such as the one used in our baseline model. Moreover, the proposed CC-trained CAP was able to generalize training and continued to perform better than the baseline classification model even when the signal-generation parameters differed or were out-of-distribution from the signals of the training dataset. We note that the generalization performance of the proposed approach was due to the fact that the CC features for distinct signals:

(13) $$\begin{array}{c} x\left(t\right)=as\left(t\right)e^{i(2\pi f_{1}t+\phi_{1})}+w_{x}\left(t\right), \end{array}$$

(14) $$\begin{array}{c} y\left(t\right)=as\left(t\right)e^{i(2\pi f_{2}t+\phi_{2})}+w_{y}\left(t\right), \end{array}$$

where $w_{x}\left(t\right)$ and $w_{y}\left(t\right)$ are independent AWGN processes and $f_{1}$, $f_{2}$, are randomly distributed (but not necessarily with the same distribution) are identical to within the measurement error, and thus, the disjoint probability density functions for the CFO in the two datasets had little effect on the classification performance. That is, the CFs differed, but the CC values were invariant to this difference.

### 5.3. I/Q-Trained CAP Performance

We also include in Figure 4 and Figure 5 plots for the $P_{CC}$ values versus the SNR for the CAPs trained using the I/Q data discussed in [23]. The performance results were similar to those reported in [23], and we note that, despite outperforming both the baseline model and the CC-trained CAP and achieving very good classification performance with signals that have similar generation characteristics as those in the training dataset, the CAP trained using the I/Q data failed to generalize and had poor performance when tested on signals coming from the alternative datasets with characteristics that had not been used in training. Thus, employing CAPs that use the I/Q signal data for training and modulation classification is not feasible for practical settings since it would only work reliably under signal conditions that fall exactly within its training dataset.

### 5.4. Confusion Matrix Results

**Figure 6:**
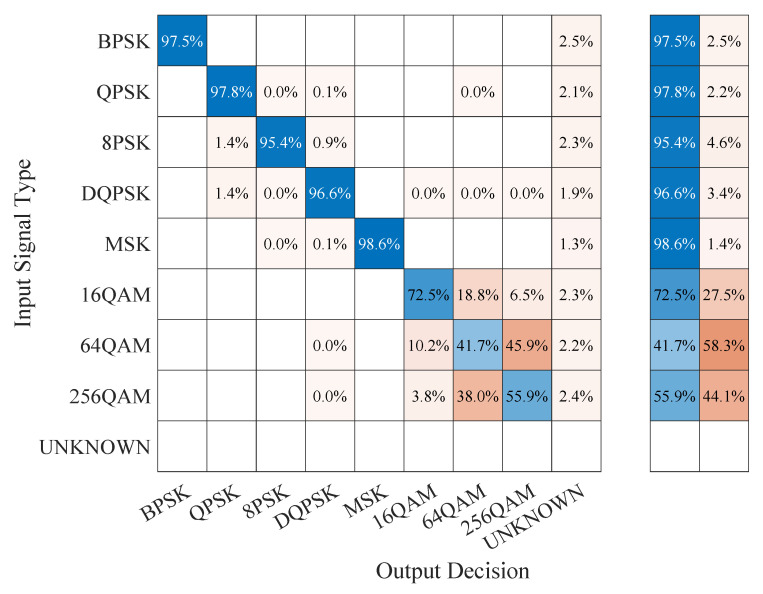
Confusion matrix of the baseline classifier on all CSPB.ML.2018 signals.

**Figure 7:**
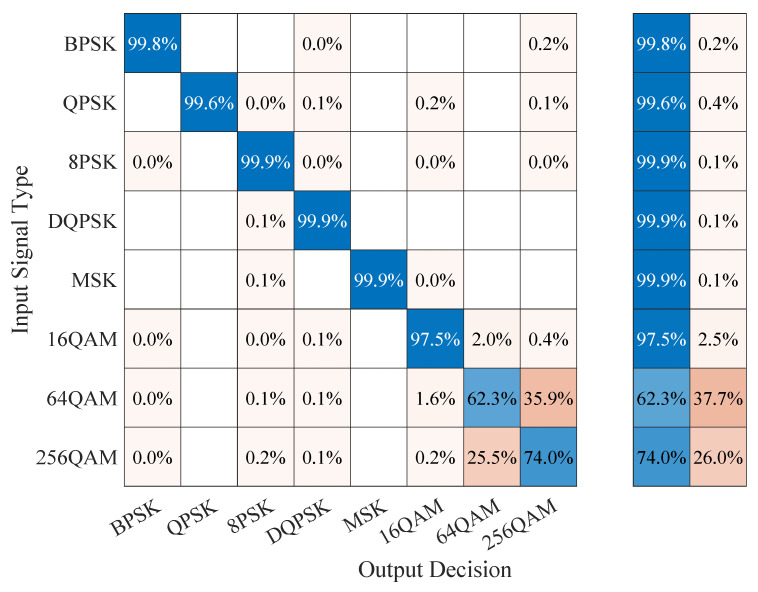
Confusion matrix of the CSPB.ML.2022 CC-trained CAP classifying all CSPB.ML.2018 signals.

**Figure 8:**
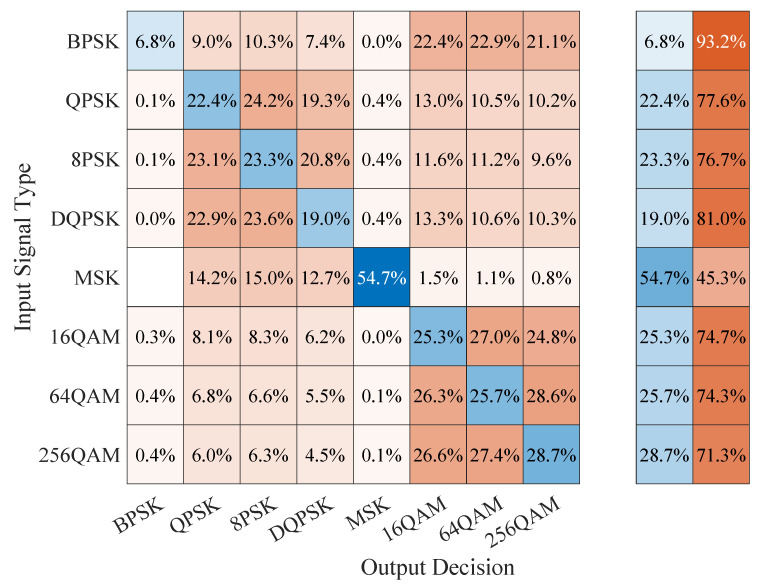
Confusion matrix of the CSPB.ML.2022 I/Q-trained CAP classifying all CSPB.ML.2018 signals.

To gain further insight into the classification performance of the proposed CAPs that use CCs for training and testing, we also looked at the corresponding confusion matrices, comparing them with those corresponding to the baseline classification model, as well as to those of the CAPs that used the I/Q data [23]. The results for the CSPB.ML.2018 dataset are illustrated in Figure 6, Figure 7 and Figure 8, from which we note that:

- The confusion matrix for the baseline classification model in Figure 6 showed that, for 5 out of the 8 digital modulation schemes of interest (BPSK, QPSK, 8PSK, DPSK, and MSK), the classification exceeded 95% accuracy, while for the remaining 3 schemes, which were all QAM-based, the classification accuracy was at 72.5% for 16QAM, 55.9% for 256QAM, and 41.7% for 64QAM. We note the “unknown” classification label, which appears in the confusion matrix of the baseline classifier because this was not trained, but rather made its classification decision based on the proximity of the estimated CCs to a modulation’s theoretical CC values as outlined in Section 2.3 and discussed in [10]. Thus, when the baseline classifier was not able to match a signal with a known pattern, it declared it “unknown” instead of confusing it with a different type of signal as DL-based classifiers do.
- For the CC-trained CAPs, we show in Figure 7 the confusion matrix corresponding to the generalization experiment, in which the capsule network was trained on the CSPB.ML.2022 dataset followed by testing using all signals in the CSPB.ML.2018 dataset. We note that the CAP showed almost perfect accuracy (exceeding 99%) for the BPSK, QPSK, 8PSK, DPSK, and MSK modulation schemes, with significant improvement over the baseline model for the remaining QAM modulation schemes, for which the classification accuracy increased to 97.5% for 16QAM, 74% for 256QAM, and 62.3% for 64QAM, which implied about 20% or more improvement over the baseline model classification performance.
- In contrast, the I/Q-trained CAP confusion matrix shown in Figure 8 corresponding to the generalization experiment (the CAP was trained on the CSPB.ML.2022 dataset followed by testing on all signals in the CSPB.ML.2018 dataset) showed very poor classification accuracy, despite having excellent accuracy when classifying the 25% test portion of the signals in the CSPB.ML.2018 dataset [23].

Similar classification accuracies were observed for the signals in the CSPB.ML.2022 dataset, but the corresponding confusion matrices were omitted from the presentation for brevity.

### 5.5. Computational Aspects

From a computational perspective, we note that both the baseline classifier and the proposed CAP-based classifier required the estimation of the CC features, for which the computational burden was variable depending on the CF pattern determined during processing (the computation was data-adaptive, unlike simpler signal-processing operations such as the FFT). When the processing is blind, as it is in this work, second- and higher-order processing is applied to find high-accuracy estimates of the rate and carrier offset. Once these key parameter values are known and the CF pattern is determined, the CC computation can commence.

The computational cost of obtaining a CC feature was determined by the following costs for the major computational steps:

1. Blind exhaustive spectral correlation and coherence analysis for *N* complex values and $N^{\prime }$ strips in the strip spectral correlation analyzer (SSCA) algorithm [36]: $NN^{\prime }\left(\log_{2}\left(N^{\prime }\right)+\log_{2}\left(N\right)+4\right)$.
2. The cost of estimating the quadrupled-carrier from the FFT of $x^{4}\left(t\right)$, and therefore the carrier offset, if the CF pattern was not determined to be BPSK-like or SQPSK-like after SSCA analysis: $3N+N\log_{2}\left(N\right)$.
3. The cost of the cyclic moments in (11) was determined by the cost of creating the needed delay products (such as $x\left(t\right)x\left(t\right)x^{*}\left(t\right)x^{*}\left(t\right)$) and the DFTs for each combination of lag product and needed cycle frequency. The number of required lag-product vectors is *P*, which was maximum for BPSK-like and minimum for 8PSK-like, where P=3. Assuming *K* CFs across all orders *n* and lag products, the cost of this step was $PN+KN\log_{2}\left(N\right)$.
4. The cost of combining the CTMFs after their computation was negligible compared to the previous sketched costs.
5. Total cost (blind processing): $NN^{\prime }\left(\log_{2}\left(N^{\prime }\right)+\log_{2}\left(N\right)+4\right)+\left(P+3\right)N+N\left(K+1\right)\log_{2}\left(N\right)$.

For example, when operating on an Intel Xeon E3-1535 laptop using C-language implementations of all operations, the total elapsed time when obtaining a BPSK CC feature for a maximum cumulant order of six, *N* = 32,768, and $N^{\prime }=64$ was 0.18 s and for the same parameters, but for an 8PSK signal, the elapsed time was 0.10 s.

Once the CC features were available, the subsequent processing to classify the signals was minimal in the context of modern processors capable of performing billions of floating-point operations per second:

- In the case of the baseline classifier, the subsequent processing involved a comparison of the estimated CC features to theoretical features to identify the closest, in terms of a distance metric, theoretical CF pattern, as outlined in Section 2.3.
- In the case of the CAP classifier, the classifier was presented with the CC feature at the input, and the classification decision corresponded to the CAP output. We note that the one-time computational cost for training the CAP should also be included in the cost in this case.

Furthermore, when comparing the proposed CAP classifier with the CAP-based classifier in [23], which uses the I/Q data of the signal to be classified, we note that the total number of trainable parameters of the latter classifier was 2,079,304 and was significantly larger than that of the proposed classifier, which only had 1,269,072 learnable parameters. This difference impacted the one-time training cost of the classifier, but once trained, the two CAPs should be able to reach rapid classification decisions.

We conclude the discussion on the computational aspects by noting that the parallel branches of the CAP are well suited for FPGA implementations, since FPGAs are designed for parallel computations. While this does not reduce the number of operations performed, it does significantly reduce the latency of calculations, which is also an important consideration in practical implementations.

## 6. Discussion

The results of this work suggested that a high degree of generalization cannot be obtained using DL-based approaches applied to the modulation-recognition problem if the input to the DL neural network is constrained to be sampled time-domain I/Q data. On the other hand, if the inputs to the DL neural networks were carefully selected features estimable from I/Q data, such as cyclic cumulants, the observed degree of generalization was very high, and the performance was also high. The fundamental research question is: Why do I/Q-based DL neural networks not generalize well? A second urgent question is: Why do I/Q-trained neural networks not learn features like cyclic cumulants?

We speculate here that the reason I/Q-trained neural networks do not learn simultaneously high-performing and high-generalization features is due to their structure and hyperparameters. Most RF domain machine learning systems (that the authors know about) have adopted the structure (layers and the order of layers) and the hyperparameters that have proven capable of performing high-quality image and natural language recognition, such as AlexNet. However, modulation recognition using sampled data is a problem that differs from image recognition in that the sought-after label is not associated with an additive component of the input; there is no BPSK part of the I/Q sequence; the whole sequence has a BPSK nature.

What separates a BPSK signal from a QPSK signal is the underlying probability density function for the transmitted symbol. That symbol random variable is binary for BPSK and quaternary for QPSK. This one density difference then leads to divergent *n*th-order probability density functions for the signal’s samples. If a neural network could be trained to learn several of these density functions from labeled data, high performance and high generalization might be obtained. However, estimating higher-order probability density functions likely requires explicit nonlinear layers rather than multiple linear convolutional layers. Therefore, we speculate that no amount of training or adjustment of the hyperparameters will lead to high generalization for a DL neural network with I/Q input; structural changes are needed.

Specific future research problems suggested by this work include:

- Investigating the performance and generalization for further datasets with more signal types and randomized multipath channels;
- Determining the $P_{CC}$ performance of the developed capsule network as a function of the input I/Q vector length. Can it reach $P_{CC}=1$? In tandem, can the baseline signal-processing method provide $P_{CC}$ near one for larger input vector lengths?
- What is the fundamental reason that DL neural networks (including capsule networks) fail to generalize with I/Q input data?
- Why do I/Q-trained DL networks not learn CC features? Can they be modified to do so by modifying the form of the feedback error and/or modifying the network layers and structure? Can the I/Q-input neural network be forced to learn CCs by imposing dimensionality or variability constraints on the latent embedding?

## 7. Conclusions

This paper presented a novel deep-learning-based classifier for digitally modulated signals that uses capsule networks and blindly estimated cyclic-cumulant features as the input. The proposed classifier outperformed conventional (non-machine-learning) classifiers employing CSP and had very good generalization abilities, unlike conventional CNNs using I/Q sample inputs, which can achieve excellent performance, but have not been made to generalize. This work, and the work upon which it was built [23,24,32], showed that the combination of conventional NNs and sampled-data inputs did not lead to both good classification performance and good generalization. The use of principled features as inputs, such as CCs, did in fact lead to simultaneous good performance and good generalization. The next step in this research is to attain that simultaneity without having to perform the signal-processing feature-extraction step outside of the network. To do this, we intend to explore new nonlinear layers in neural networks, de-emphasizing convolutions and, thereby, regaining the convenience of using sampled data inputs while retaining the performance and generalization associated with the principled statistical features.

## Figures and Tables

**Table 1:** CC-trained CAP layout.

Layer	(# of Filters)[Filt Size]	Stride	Activations
Input			11×15×1
Conv	(56)[6×4×1]	[1×2]	11×8×56
Batch Norm			
Tanh			
Conv-1-(i)	(56)[4×4×56]	[1×2]	11×4×56
Batch Norm-1-(i)			
Tanh-1-(i)			
Conv-2-(i)	(72)[4×6×56]	[1×2]	11×2×72
Batch Norm-2-(i)			
Tanh-2-(i)			
FC-(i)			7
Batch Norm-3-(i)			
ReLu-1-(i)			
Point FC-(i)			1
Depth Concat (i = 1:8)			8
SoftMax			

**Table 2:** Dataset signal-generation parameters.

Parameter	CSPB.ML.2018	CSPB.ML.2022
Sampling Frequency, $f_{s}$	1 Hz	1 Hz
Carrier Frequency Offset	uniformly	uniformly
(CFO), $f_{0}$	distributed in	distributed in
	(−0.001,0.001)	(0.01,0.02)
Symbol Period, $T_{0}$, Range	[1,23]	[1,29]
SRRC Pulse-Shaping		
Roll-Off Factor, β, Range	[0.1,1]	[0.1,1]
In-Band SNR Range (dB)	[0,12]	[1,18]
In-Band SNR Center of Mass	9 dB	12 dB

**Table 3:** Classification performance.

Classification Model	Results for Dataset CSPB.ML.2018	Results for dataset CSPB.ML.2022
Baseline Model	82.0%	82.0%
CSPB.ML.2018 I/Q-trained CAP	97.5%	23.7%
CSPB.ML.2022 I/Q-trained CAP	25.7%	97.7%
CSPB.ML.2018 CC-trained CAP	92.3%	93.1%
CSPB.ML.2022 CC-trained CAP	91.6%	92.5%

## Data Availability

The datasets used to obtain the numerical results presented in this paper are openly available from the IEEE DataPort and the CSP Blog [34].
